# Towards a comprehensive regulatory map of Mammalian Genomes

**DOI:** 10.21203/rs.3.rs-3294408/v1

**Published:** 2023-09-28

**Authors:** Tássia Mangetti Gonçalves, Casey L Stewart, Samantha D Baxley, Jason Xu, Daofeng Li, Harrison W Gabel, Ting Wang, Oshri Avraham, Guoyan Zhao

**Affiliations:** Washington University School of Medicine; University of Georgia; University of Georgia; Missouri University of Science & Technology; Washington University School of Medicine; Washington University School of Medicine; Washington University School of Medicine; University of Georgia; Washington University School of Medicine

**Keywords:** transcription factor, positive weight matrix, enhancers, Cis-regulatory modules, epigenomics, machine learning

## Abstract

Genome mapping studies have generated a nearly complete collection of genes for the human genome, but we still lack an equivalently vetted inventory of human regulatory sequences. Cis-regulatory modules (CRMs) play important roles in controlling when, where, and how much a gene is expressed. We developed a training data-free CRM-prediction algorithm, the Mammalian Regulatory MOdule Detector (MrMOD) for accurate CRM prediction in mammalian genomes. MrMOD provides genome position-fixed CRM models similar to the fixed gene models for the mouse and human genomes using only genomic sequences as the inputs with one adjustable parameter – the significance p-value. Importantly, MrMOD predicts a comprehensive set of high-resolution CRMs in the mouse and human genomes including all types of regulatory modules not limited to any tissue, cell type, developmental stage, or condition. We computationally validated MrMOD predictions used a compendium of 21 orthogonal experimental data sets including thousands of experimentally defined CRMs and millions of putative regulatory elements derived from hundreds of different tissues, cell types, and stimulus conditions obtained from multiple databases. *In ovo* transgenic reporter assay demonstrates the power of our prediction in guiding experimental design. We analyzed CRMs located in the chromosome 17 using unsupervised machine learning and identified groups of CRMs with multiple lines of evidence supporting their functionality, linking CRMs with upstream binding transcription factors and downstream target genes. Our work provides a comprehensive base pair resolution annotation of the functional regulatory elements and non-functional regions in the mammalian genomes.

## Background

Transcriptional regulation ensures that every gene is transcribed at the right time, in the right cells, and in the right amount^1^. Cis-regulatory modules (CRMs) are defined as DNA sequences with transcription factor binding sites (TFBSs) clustered into modular structures to regulate spatiotemporal gene expression^2^ and include enhancers, promoters, locus control regions, silencers, and other modulators. CRM disruption has been implicated as a disease-driving mechanism for many diseases such as cancer^3,4^ and neurological disorders^5^. Despite their clear importance to both basic and disease biology, we still lack a complete understanding about the repertoire of human enhancers, including where they reside, how they work, and what genes they mediate their effects through^6–8^.

CRM discovery methods can be broadly classified into two categories: empirical and computational approaches, although in reality the two are often intertwined^1^. Classically, CRMs were defined through functional assays—primarily reporter gene assays—that demonstrate the ability of a given sequence fragment to affect transcription. Those experimentally defined CRMs (ExpCRMs) still serve as gold standards of functional CRMs. However, these approaches are low-throughput, expensive, and time-consuming^1,9^. VISTA Enhancer Browser is the only database that has a collection of mammalian enhancers experimentally defined in transgenic mice^10,11^ with 673 and 998 from the mouse and human genomes, respectively. Recently, high-throughput empirical approaches such as chromatin immunoprecipitation coupled to sequencing (ChIP-seq)^12,13^, DNase I hypersensitive sites (DHS) sequencing (DNase-seq), transposase-accessible chromatin using sequencing (ATAC-seq)^14,15^, STARR-seq^16^, and massively parallel reporter assay (MPRA)^17^, have greatly facilitated candidate enhancer identification on a genome-wide scale^4^. Despite the enormous amount of data generated by these methods however, empirical methods only identify subsets of potential active enhancers in the genome because it is currently not possible to sample every cell type, every developmental stage, or every environmental stimulus due to limited availability of reagents, starting materials, and technologies^7^. DNA fragments defined by these methods are “putative enhancers” and should be validated through gold-standard experimental approaches to provide additional evidence of function^8,13^. Additionally, because no reference CRM models analogous to gene models exist, peak calling is required in epigenomic data analyses to first define the genomic regions with the epigenomic signal of interest. It is common that different peaks or peaks with different positions and lengths are called in biological replicates and the number of peaks can vary hugely depending on the program used for peak calling^18^. This makes signal comparison across biological replicates and conditions much more challenging than comparing gene expression using the RNA-seq method, which has a well-annotated set of gene models as references^19^. Computational methods for CRM discovery can be broadly classified into three main categories depending on the types of input data they require. 1) The comparative genomics approach depends on identifying conserved non-coding DNA sequence regions across species under the assumption that functionally important genomic sequences are under more evolutionary constraint than sequences with less-vital functions. However, not all CRMs are conserved and this type of method fails to discover newly evolved regulatory modules^1,7^. 2) Motif-based methods (reviewed in^1,7^) rely on the identification of clusters of TFBSs, requiring prior knowledge of the constituent motifs, to predict new CRMs with similar features. 3) “Motif-blind” approaches are based on properties of the input sequences such as known CRMs or epigenomic datasets to predict functional CRMs (reviewed in^20^). These categories are not mutually exclusive, and methods combining multiple approaches often perform strongly. However, most of them require a set of known CRMs, TF binding information, or epigenomic data as training data^1,7,20^. Due to the limited knowledge of known TFs and functional CRMs, and the limitation of currently available epigenomic data, none of the current methods can detect all types of CRMs for all cell types, developmental stages, and conditions.

To overcome the limitations of the current computational CRM discovery methods, we developed the Mammalian Regulatory MOdule Detector (MrMOD) to accurately predict CRMs in mammalian genomes. MrMOD started with the mouse, rat, and human genomes and has several unique features compared to current methods. 1) There is no requirement of training data; 2) It has only one parameter to adjust: p-value < 0.01, one-tailed; 3) It predicts a variety of currently known types of regulatory modules; 4) It provides a comprehensive set of high-resolution CRMs (average size < 220 bp) not limited to any tissue, cell type, developmental stage, or stimulus condition; 5) The predicted CRMs are final, providing a fixed position for every CRM (refCRM) in the genome similar to the fixed positions for every gene in the genome. We used multiple sources of orthogonal data to evaluate predicted CRMs including thousands of ExpCRMs collected from the literature and VISTA Enhancer Browser, 19 epigenomic data sets that represent putative regulatory elements from Enhancer Atlas^21^, ENCODE cCREs^22^, Cistrome Data Browser^23^, as well as single-cell ATAC-seq data (scATAC-seq) from the mouse brain atlas^24^ and human genome^25^ derived from hundreds of cell types, tissues, developmental stages, and conditions. Comparing a subset of the predicted functional CRMs with genomic regions that had the exact same length distribution and genomic coverage but predicted to be non-functional achieved high sensitivity and odds ratio demonstrating the accurate prediction of functional regulatory sequences. Experimental dissection of a large enhancer from the VISTA database guided by our predicted CRMs delineated smaller enhancers with more restricted spatiotemporal expression. To annotate the functionality of predicted CRMs, we scanned the mouse genome for TFBSs using the Position weight matrices (PWMs, models the binding specificity of TFs)^26^ in the Catalog of Inferred Sequence Binding Preferences (CIS-BP) databases^27^. Unsupervised machine learning defined groups of CRMs with similar motif compositions, the TFs that interact with the CRMs, and the putative target genes regulated by the set of TF-CRMs. We provide one example from the mouse chromosome 17 with multiple lines of evidences supporting their roles in the regulation of gene expression in cancer, neurodevelopmental, and neurodegenerative disorders. We provide access to the predicted CRMs and the experimental supporting evidence across the human and mouse genomes as part of the WashU Epigenome Browser.

## Results

### Genome-wide TFBS prediction and validation in the mammalian genomes

**Figure 1:**
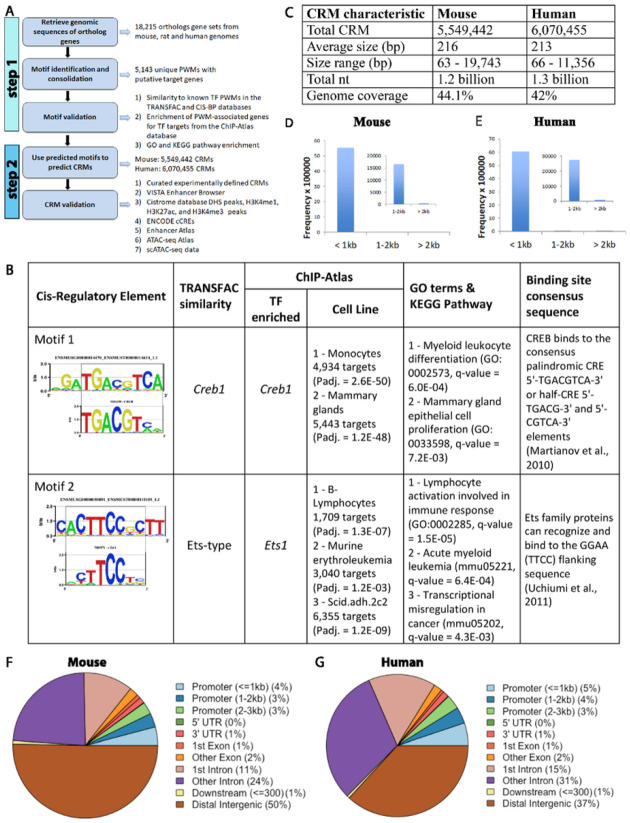
Whole-genome-wide cis-regulatory module (CRM) prediction and validation in mammalian genomes. A) A schematic diagram of workflow of whole-genome-wide CRM prediction. In step 1, we defined a set of non-redundant conserved TFBS motifs in the mammalian genomes via applying PhyloNet on the genomic sequences of mouse, rat, and human. In step 2, we applied the algorithm implemented in CerMOD on the genomic sequences of human and mouse using the set of mammalian TFBS motifs obtained from the first step to predict mammalian CRMs. **B**) Examples of predicted PWMs and supporting evidence. For both motif 1 and motif 2, the top logos represent the predicted PWMs and the logos below represent the binding motifs of a known TF from the TRANSFAC database. **C**)Summary statistics of mouse and human complete set of predicted CRMs (CRMfull). **D**) Mouse CRMfull length distribution. **E**) Human CRMfull length distribution. **F, G**) The distributions of CRMfull relative to the genomic features for the mouse (**F**) and human (**G**) genome respectively.

The workflow of whole-genome-wide CRM prediction includes two steps (Fig. 1A). First, we defined a set of non-redundant conserved TFBS motifs in the mammalian genomes via applying PhyloNet^28^ on the genomic sequences of mouse, rat, and human using published workflow^29^. Second, we applied the algorithm implemented in CERMOD^29^ on the genomic sequences of human and mouse using the set of mammalian TFBS motifs obtained from the first step to predict mammalian CRMs. PhyloNet systematically identifies phylogenetically conserved motifs that also occur multiple times throughout the genome to define a network of regulatory sites for a given organism^28^. A final set of 5,143 unique PWMs (p-value < 10^− 10^) with an average length of 18 bp (ranging from 5 to 30 bases) was obtained after consolidation of redundant motifs as described^29^. Each predicted PWM is associated with a set of genes that are potentially regulated by this motif.

Next, we performed multiple analyses to assess the functionality of the predicted PWMs. 1) We compared the predicted PWMs to known TF PWMs in the TRANSFAC^30^ and the CIS-BP databases^27^ using the average log likelihood ratio (ALLR) statistic^31^ and OLAP score as described^29^ (Table 1). Out of the 5,143 predicted PWMs, 836 (16.3%) and 2,203 (42.8%) shared similarity with at least one PWM from the TRANSFAC and CIS-BP database, respectively. 2) We tested whether each PWM-associated gene set was enriched for TF targets determined in the ChIP-Atlas database^32^, which has a collection of 2,540 ChIP-seq datasets including 723 mouse TFs in 369 different cell types and tissues. A total of 637 (12.4%) PWM-associated gene sets were significantly enriched for known TF targets in at least one condition (hypergeometric test, false discovery rate (FDR) corrected p-value < 0.05). 3) We tested whether the set of PWM-associated genes was enriched for specific classes of genes using Gene Ontology (GO) and KEGG pathway enrichment analyses. We found that 2,678 (52%) PWMs had significant enrichment for at least one GO term and 614 (12%) PWMs had significant enrichment for at least one KEGG pathway. We observed multiple lines of evidence from different categories consistent with the known functions of corresponding TFs which provides strong support that the majority of the PhyloNet-discovered PWMs represent the DNA-binding specificity for the corresponding TFs (Fig. 1B, Table 1).

### Whole-genome-wide CRM prediction

Next, we developed MrMOD to perform whole-genome-wide CRM predictions (referred to as “CRMfull” herein) for mouse and human genomes using the CERMOD^29^ algorithm but with the set of 5,143 unique mammalian PWMs obtained from the first step as the input. MrMOD predicted a total of 5,549,442 (44.1% of genome coverage) and 6,070,455 CRMs (42% genome coverage) with an average size of 216 and 213 bp for the mouse and human genomes, respectively (Fig. 1C). The length ranged from 63 to 19,743 bp but the majorities were smaller than 1 kb for both genomes (99.7% and 99.6%, Fig.1D). The majority of the predicted CRMs are located within distal intergenic regions or intronic regions with about 10% located within 3 kb of a promoter for both human and mouse genomes (Fig. 1F, 1G), similar to that observed for the human putative regulatory elements defined using DNase I hypersensitive sites (DHSs)^14^.

### Obtaining control regions for the evaluation of CRM predictions

**Figure 2:**
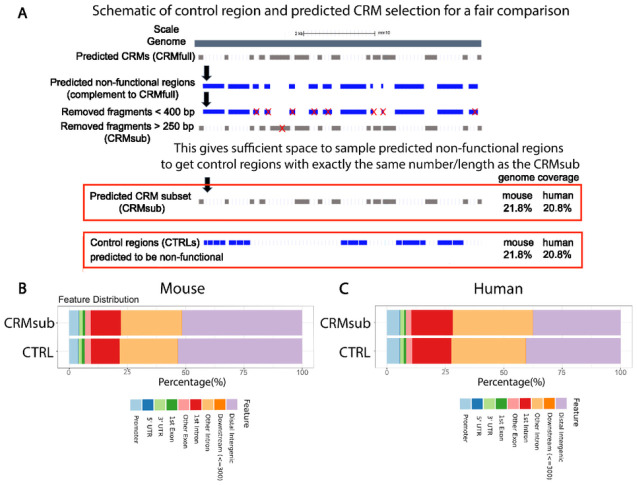
Obtaining control regions for a fair evaluation of whole-genome-wide CRM predictions. **A)** Schematic of control region and predicted CRM selection for a fair comparison. **B-C**) Comparison of the distributions between CRMsub and CTRLs relative to the genomic features for the mouse (**B**) and human (**C**) genome respectively.

To systematically evaluate the accuracy of predicted CRMs we need to obtain a set of control regions that are not enriched for regulatory functions. However, there is no collection of experimentally defined non-functional regions. Therefore, we devised a scheme to obtain a set of control regions that have the exact same length distribution and genomic coverages as a subset of CRMs from each genome but are predicted to be non-functional for a fair comparison (Fig. 2A, details in the Materials and Methods section). First, we obtained regions not covered by any predicted CRMs, which correspond to regions predicted to be non-functional. Because it is difficult to predict the exact boundaries of CRMs, small DNA fragments representing regions located between two predicted CRMs were removed, as they could be part of the nearby functional CRMs. The predicted non-functional regions did not have sufficient space to generate the exact length distribution of the full-predicted CRM set. Therefore, we gradually eliminated the longest CRMs to obtain a predicted CRM subset (CRMsub) such that we can generate a set of control regions with the same number and length distribution located within the predicted non-functional regions (referred to as CTRLs) for performance evaluation. The CRMsub and the CTRLs had the exact same genomic coverage and distributions relative to genomic features for both genomes (Fig. 2B, 2C), with 21.8% and 20.8% of genomic coverage for mouse and human, respectively. Therefore, the CTRL regions provided good data sets for a fair performance evaluation.

### Predicted CRMs include diverse classes of functional regulatory sequences

To evaluate the accuracy of predicted CRMs we performed an extensive literature search to identify any mouse or human genes whose genomic regions have been analyzed using *in vitro or in vivo* reporter assays to locate any regulatory sequences (referred to as experimentally defined CRMs, or ExpCRMs). Wherever possible, we used regions that were determined by an enhancer assay using minimal sequence sufficient to drive reporter gene expression because it better defines the boundaries of regulatory regions that are sufficient in regulation^29^. Only ExpCRMs smaller than 2.5 kb were included in the analyses because > 90% of the peaks in all are smaller than 2.5 kb datasets (except VISTA Enhancer Browser). For mouse, we collected 97 ExpCRMs associated with 56 genes including enhancers, silencers, insulators, locus control regions, and RNA post-transcriptional regulatory modules that regulate gene expression in a diverse cell types, tissues, and developmental stages, covering a total of 54,624 bp with an average size of 563 bp (range 11 to 2,000 bp, Supplementary Table 1). Similarly, a total of 60 ExpCRMs associated with 43 human genes were collected with 10 CRMs defined by mouse *in vivo* reporter assays and 50 by *in vitro* assays covering 20,610 bp with an average size of 343 bp (range 25 to 1,515 bp, Supplementary Table 2). For our evaluation of accuracy an ExpCRM was deemed as correctly predicted if the predicted CRM and ExpCRM overlap at least 50% of the length of the smaller fragment or if they overlap at least 1 bp (referred to as 50%- or 1bp-overlap cutoff herein).

#### 1) Accurate prediction of enhancers and silencers:

**Figure 3:**
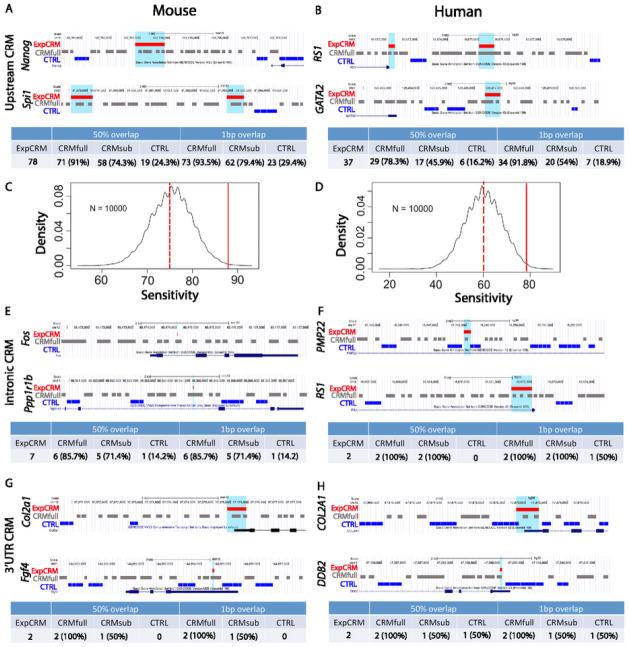
Comparison of predicted CRMs with experimentally defined modules located in the upstream intergenic regions, introns, and 3’ UTRs. **A-B)** Comparison between predicted CRMfull, CRMsub, and CTRLs with experimentally defined CRMs (ExpCRMs) located within 50 kb upstream of ATG start codon using **A**) mouse *Nanog* and *Spi1*, and **B**) human *RS1* and *GATA2* as examples. **C**) Comparison of sensitivity with simulated data for mouse data (repeated 10,000 times). The mouse average sensitivity is 75.4% (dashed red line). The solid vertical red line represents the real sensitivity of mouse predictions (88.4%). **D**) Comparison of sensitivity with simulated data for human data (repeated 10,000 times). The human average sensitivity is 60.1% (dashed red line). The solid red line represents the real sensitivity of human predictions (78.3%). **E-F**) Comparison between CRMfull,CRMsub, and CTRLs with ExpCRMs located in the intronic regions of **E**) mouse *c-fos* and *Ppp1r1b* and **F)** human *PMP22* and *RS1*. **G-H**) Comparison between CRMfull, CRMsub, and CTRLs with ExpCRMs located in the 3’ UTR of **G**) mouse *Col2a1* and *Fgf4* and **H**) human *COL2A1* and *DDB2*. Red bar: experimentally defined CRMs; gray bar: predicted CRMs; blue bar: CTRL regions; dark blue box and line: gene models.

We first evaluated MrMOD prediction accuracy using curated ExpCRMs that are enhancers or silencers located in the intergenic regions upstream of ATG start codons, the intronic sequence, or the 3’ UTRs. **1.1) Accurate prediction of upstream CRMs**: We defined modules that were located upstream of the ATG start codon and downstream of the next nearest gene as upstream CRMs. We used ExpCRMs that were located within 50 kb upstream of ATG in both genomes (69 mouse and 37 human ExpCRMs) and compared them to the predicted CRMs located within the same genomic ranges. Figure 3A shows an example comparing predicted CRMs with ExpCRMs located upstream of the Nanog homeobox gene (*Nanog*) and Spi1 proto-oncogene (*Spi1*) in the mouse genome. The *Nanog* enhancer drives gene expression in early mesoderm-specified progenitors^33^. *Spi1* has two CRMs previously reported in the literature with one driving reporter gene expression in transgenic mouse at embryonic sites of the endothelial-to-hematopoietic transition and the other driving non-hematopoietic gene expression^34^. All three ExpCRMs were correctly predicted without overlapping with the CTRL regions. We have a total of 78 mouse ExpCRMs that are upstream the ATG but not limited to 50kb within the ATG. A total of 71 out of 78 ExpCRMs (91%) were correctly predicted whereas only 19 (24.3%) overlapped with the CTRL regions at 50%-overlap cutoff. Because CRMfull has higher genomic coverage than CTRL regions, this comparison is not suitable for statistical testing. Therefore, we performed simulations by randomly shuffling the positions of predicted CRMs within each sequence to estimate the statistical significance of obtaining the given sensitivity. Using mouse ExpCRMs within 50 kb upstream of ATG the average sensitivity of simulated CRMs is 75.4% with a standard deviation of 4.4 for 10,000 simulations (Fig. 3C), demonstrating high sensitivity (88.4%, p-values < 0.01) in MrMOD predictions for mouse CRMs located within 50kb of ATG (upstream regions). Similarly, all three enhancers upstream of the human GATA binding protein 2 (*GATA2*)^35^ and retinoschisin 1 (*RS1*)^36^ were correctly predicted without overlapping with the CTRL regions (Fig. 3B). A total of 29 (78.3%) out of the 37 ExpCRMs overlapped with CRMfull whereas only 6 (16.2%) overlapped with the CTRL regions. Simulated CRMs had an average sensitivity of 60.1% with standard deviation of 7.7 (Fig. 3D, p-values < 0.01 for a sensitivity of 78.3%). We cannot calculate the positive predictive value because the majority of the predicted modules are located within DNA sequences that have not been tested, or DNA fragments drive reporter gene expression in additional tissues but location information was not reported^37^. **1.2) Accurate prediction of intronic CRMs**: Fig. 3E demonstrates that both the mouse c-*fos*^38^ and the protein phosphatase 1 regulatory inhibitor subunit 1B (*Ppp1r1b*) intronic enhancers (11 and 23 bp, respectively)^39^ overlapped with predicted CRMs but not the CTRL regions. In total, 6 out 7 (85.7%) intronic mouse ExpCRMs were correctly predicted and only one overlapped with the CTRL regions. We collected two human intronic ExpCRMs and both overlapped with predicted CRMs but not the CTRL regions at 50%-overlap cutoff (Fig. 3F). **1.3) Accurate prediction of CRMs located in the 3’ UTR regions**: We collected two mouse enhancers located in the 3’ UTR of *Fgf4* (64 bp)^40^ and *Col2a1* (543 bp)^41^. Both were correctly predicted without overlapping with the CTRL regions at either cutoff (Fig. 3G). The two curated human ExpCRMs located in the 3’ UTR of *COL2A1*^41^ (537 bp) and *DDB2* (49 bp)^42^ were also correctly predicted (Fig.3H).

#### 2) Accurate prediction of putative post-transcriptional regulatory elements

**Figure 4:**
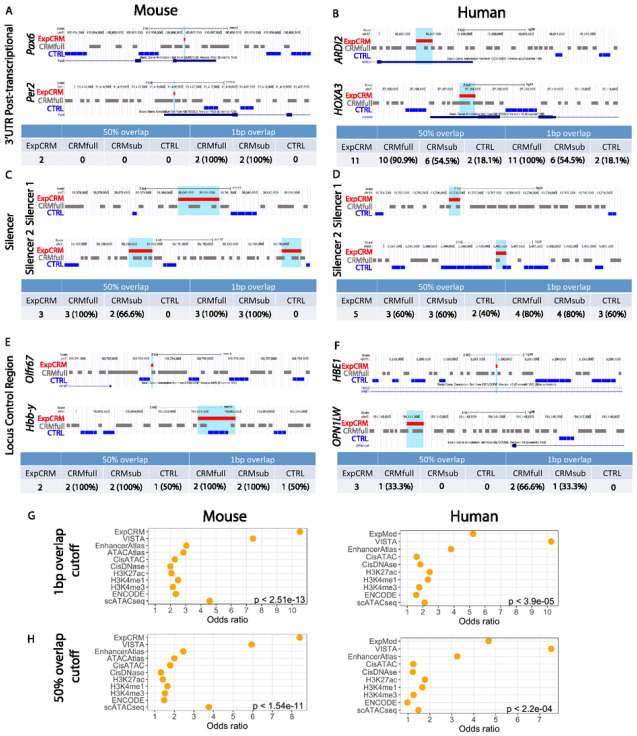
Comparison of predicted CRMs with experimentally defined modules (ExpCRMs) with different functionality and statistical evaluation of the whole-genome-wide CRM predictions. **A-B)** Comparison between CRMfullwith the ExpCRMs with post-transcriptional regulatory function located in the 3’ UTRs of **A**) mouse *Pax6* and *Per2* and **B**) human *ARID2* and *HOXA3*. **C-D**) Comparison between CRMfull and predicted CRMs with silencer function for **C**) the three mouse *Cebpa* silencers and **D**) the two human silencer elements. **E-F**)Comparison between CRMfull with E) mouse LCR of *Olfr67* (3’ HS1) and *Hbb-y* (5’HS2) and **F**) human LCR of *HBE1*(5’ HS5) and *OPN1LW*. **G-H**) Odds ratios and p-values comparing the sensitivity between CRMsuband CTRLs at 1bp-overlap cutoff (**G**) or 50%-overlap cutoff (**H**) for mouse and human genomes. Red bar: experimentally defined CRMs; gray bar: predicted CRMs. Blue bar: CTRL regions; dark blue box and line: gene models.

Post-transcriptional gene regulation controls the amount of proteins produced from an mRNA by altering rates of decay and translation. A 37 bp miRNA recognition element (MRE) of miR-375 in mouse *Pax6* 3’ UTR was previously reported to be active in αTC1–6 cells^43^ and a 43 bp miR-24/miR-30a binding site in the mouse *Per2* 3’ UTR was reported to be functional in NIH 3T3 cells^44^. Both post-transcriptional regulatory elements overlapped with predicted CRMs at 1bp-overlap cutoff but not with CTRL regions (Fig. 4A). We collected 11 human elements validated functionally using a luciferase reporter assay^45^. All (100%) overlapped at least 1 bp with our predicted CRMs and only two of them also overlapped with the adjacent CTRL regions (Fig. 4B). These results demonstrate that our method can identify post-transcriptional regulatory elements with high sensitivity and accuracy. Because miRNA sites are not included in the sequences used for PWM identification, our predicted CRMs may also represent novel transcriptional elements that happen to overlap with miRNA sites as comparative genomic studies suggest that many additional novel regulatory elements may exist in the mammalian 3’ UTRs^45^.

#### 3) Accurate prediction of silencers

Silencer elements are undercharacterized in mammalian genomes and can be hard to predict. We therefore evaluated the sensitivity of out method for detecting silencers. Three silencers (771–979 bp) that repress the activity of the *Cebpa* proximal promoter in mouse G1ME cells^46^ were all correctly predicted at both cutoffs without overlapping with CTRL regions (Fig. 4C). Of five human silencers (301 bp) that were previously detected in K562 cells^47^, MrMOD correctly predicted four (80%) of them at 1bp-overlap cutoff with 3 of them also overlapping with adjacent CTRL regions (Fig.4D).

#### 4) Accurate prediction of locus control regions (LCRs):

Two mouse LCRs at the β-globin loci^48^ were both correctly predicted (Fig. 4E) although one also overlapped with the nearby CTRL regions. We collected three human LCRs: two (both 72 bp) that demonstrated enhancer-blocking activity^48^ and one upstream of *OPN1LW* (592 bp) essential for the expression of cone pigment genes^49^. Two were correctly predicted at 1bp-overlap cutoff and none of them overlapped with CTRL regions (Fig. 4F).

In summary, our predicted CRMs detected diverse classes of functional regulatory elements currently known in the human and mouse genomes with high sensitivity. Many of the ExpCRMs that overlapped with predicted non-functional CTRL regions also overlapped with predicted CRMs. For example, of the 22 mouse ExpCRMs that overlapped with CTRL regions at 50%-overlap cutoff, 19 (86.3%) also overlapped with nearby predicted CRMs. This is largely due to the large size of some ExpCRMs, which may include both functional and non-functional sequences. Further analyses are needed to identify the minimal functional sequences, which will improve accuracy of performance evaluation.

### Statistical evaluation of MrMOD predictions using multiple orthogonal datasets

To perform statistical evaluation of MrMOD predictions we used a compendium of experimentally defined enhancers and large-scale epigenomic datasets representing putative regulatory elements downloaded from multiple databases including VISTA Enhancer Browser^10^, ENCODE cCREs^22^, the Enhancer Atlas 2.0^21^, Cistrome Data Browser^23^, ATAC-seq Atlas^50^, and scATAC-seq data for mouse brain and 45 adult/fetal human tissue types^24,25^ (Supplementary Tables 3 and 4). Peak overlap between each dataset and CRMsub or CTRL regions was determined separately and peak-detection sensitivity and odds ratios (OR) were calculated comparing CRMsub to CTRL. Below is a description of all the datasets (11 for mouse and 10 for human) used in the analyses.

1. A total of 157 literature-curated mouse (97) and human (60) ExpCRMs driving gene expression in a diverse type of tissues, cell types, and developmental stages.
2. A total of 1,632 *in vivo* validated functional enhancer elements for mouse (634) and human (998) from VISTA Enhancer Browser
3. Enhancer Atlas 2.0 contains a total of 530,094 and 201,032 putative enhancers for mouse and human, respectively, annotated in 534 different tissues/cell types^21^.
4. DHS peaks: DHS regions contain a variety of regulatory elements including enhancers, silencers, insulators, and locus control regions^22^. Mouse (1,242,162 peaks) and human (2,401,388 peaks) DNase-seq data were downloaded from Cistrome database.
5. Bulk ATAC-seq peaks: The Cistrome database contained 1,124,888 mouse ATAC-seq peaks derived from 1,937 samples and 46 different tissues and cell types. The human ATAC-seq data contained 1,217,002 peaks from 1,059 samples and 25 different tissues and cell types. Additionally, the Mouse ATAC-seq Atlas database has a total of 296,390 peaks derived from 66 ATAC-seq profiles from 20 primary tissues of adult mice.
6. ChIP-seq data of the histone marks H3K4me1 and H3K27ac represent putative active enhancers. Mouse H3K4me1 (1,095,988 peaks), H3K27ac (1,400,986), and human H3K4me1 (1,565,782) and H3K27ac (1,697,483) ChIP-seq data were downloaded from the Cistrome database.
7. ChIP-seq data of the H3K4me3, which represents putative promoters, were downloaded from the Cistrome database with 1,120,149 peaks for mouse and 2,081,430 peaks for human.
8. scATAC-seq detects cell-type-specific open chromatin regions potentially representing cell-type-specific regulatory elements functioning in hundreds of cell types from different tissues and developmental stages. Mouse scATAC-seq brain atlas identified 491,818 elements from 45 brain regions and 160 cell types in adult mouse cerebrum^24^. Human scATAC-seq data provided ~ 1.2 million elements from 45 adult/fetal tissues and 222 cell types^25^.
9. ENCODE cCREs are candidate cis-Regulatory Elements (cCREs) obtained through the integration of ENCODE epigenomics data, providing 368,121 cCREs in 169 tissues/cell types and 1,063,878 cCREs in 1,518 tissues/cell types for mouse and human genomes, respectively^22^.

We first used all collected ExpCRMs to evaluate CRMsub and CTRL regions. For the 97 mouse ExpCRMs, CRMsub has a detection sensitivity of 79.3% (77/97) whereas the CTRL region detection has a sensitivity of 28.8% (26/97) resulting an OR of 10.51 (p-value < 2.5e-13) at 1bp-overlap cutoff (Fig. 4G, Table 2). At a more stringent 50%-overlap cutoff, fewer CRMsub (71.1%) and CTRLs (22.6%) overlapped with the ExpCRMs but the OR remained high (8.40, p-value < 1.5e-11, Table 2, Fig.4H). For the 60 human ExpCRMs, the CRMsub has a detection sensitivity of 56.6% (34/60) whereas the CTRL region detection has a sensitivity of 20% (12/60) giving an OR of 5.23 (p-value < 3.9e-05, Fig.4G, Table 3) at 1bp-overlap cutoff. Using the more stringent 50%-overlap cutoff resulted in similar results (OR = 4.67, p-value < 2.2e-04, Fig.4H). If we use 1bp-overlap as the cutoff, the full set of predicted CRMs (CRMfull) have a detection sensitivity of 93.8% (91/97) and 90% (54/60) for mouse and human datasets, respectively (Tables 2 and 3), demonstrating highly sensitive and accurate CRM predictions made by MrMOD.

Next, we used functional enhancers from the VISTA Enhancer Browser database to evaluate our predicted CRMs. Although VISTA enhancers are also experimentally defined modules, they are much bigger than our curated ExpCRMs (average size = 2,461 vs. 541 bp for mouse) (Supplementary Table 3). Many VISTA enhancers drive reporter gene expression in multiple tissues or brain regions without cell-type specificity information. Therefore, DNA fragments in the VISTA enhancer database likely contain multiple enhancers as well as non-functional sequences between those enhancers, which could be delineated through more detailed analysis. With these limitations a high percentage of CTRL regions (68.5% and 61.2% at 50%- overlap cutoff and 71.9% and 62.6% at 1bp-overlap cutoff for mouse and human, respectively) overlapped with VISTA enhancers. However, a much higher percentage of CRMsub (92.8% and 92.2% at 50%-overlap cutoff and 95% and 94.5% at 1bp-overlap cutoff for mouse and human, respectively) overlapped with VISTA enhancers, resulting high ORs for all comparisons (5.94–10.24, p-value < 3.7e-15, Fig. 4G–H, Tables 2 and 3). If we use 1bp-overlap as the cutoff for correct prediction, our full prediction (CRMfull) has a sensitivity of 100% and 99.5% for mouse and human datasets, respectively. Again, these results demonstrate highly sensitive and accurate CRM prediction made by MrMOD.

Lastly, we used multiple epigenomic profiling datasets to evaluate our predicted CRMs. For the mouse genome, there were 9 datasets containing 296,390– 1,400,986 total peaks per dataset with an average size ranging from 276 bp to 947 bp (Supplementary Table 3). When comparing the overlaps between the collected peaks and our mouse predicted CRMsub and CTRL regions, CRMsub always had a much higher sensitivity (range 39.9%–82.3%) than did the CTRL regions (range 31%–50.3%) with ORs ranged 1.31–4.58 (p-value < 2.2E-16) at either cutoff (Fig. 4G–H, Table 2), highlighting the significant enrichment of functional elements in our predicted CRMsub compared to the CTRL regions. We obtained similar results using all epigenomic datasets to evaluate human predicted CRMs. There were 8 datasets containing 201,032 − 2,401,388 total peaks per dataset with an average size ranging from 273 bp to 952 bp (Supplementary Table 4). Our human predicted CRMsub had a much higher sensitivity (range 35.8%–74.2%) than did the CTRL regions (range 36.4%–49.8%) at either cutoff. All ORs were above 1.21 (Fig.4G–H, Table 3) except the ENCODE cCREs dataset, which has an OR of 0.97 at 50%-overlap cutoff but a much higher OR (1.57) at 1bp-overlap cutoff. This is likely due to the high resolution of predicted ENCODE cCREs (average length of 276 bp and 273 bp for mouse and human, respectively) and our predicted CRMs (216 bp for mouse and 213 bp for human).

We also evaluated MrMOD prediction using an alternative scheme of obtaining control regions (Supplementary Fig. 1A-C). In this method, the control regions (predicted non-functional regions) were longer and had slightly higher genomic coverage than the predicted CRM subset to give them an advantage in comparisons. However, we obtained results similar as described above, demonstrating high prediction accuracy and consistent performance (Supplementary Fig.1D-E, Supplementary Tables 5 and 6). Notably, our prediction had the highest ORs (5.91–16.32) compared to control regions when evaluated using curated ExpCRMs and VISTA enhancers, the “gold standard” functional regulatory sequences no matter what cutoff or control regions were used. In summary, the consistency in prediction sensitivity and OR comparing CRMsub with the CTRL regions for both mouse and human genomes across all datasets, representing diverse regulatory elements obtained from thousands of samples and derived from hundreds of different tissues and cell types at different developmental stages and conditions, suggests that our predicted CRMs represent all types of regulatory elements tested here unlimited by tissue, cell type, developmental stage, or stimulus.

### Unsupervised machine learning for CRM functional annotation

**Figure 5:**
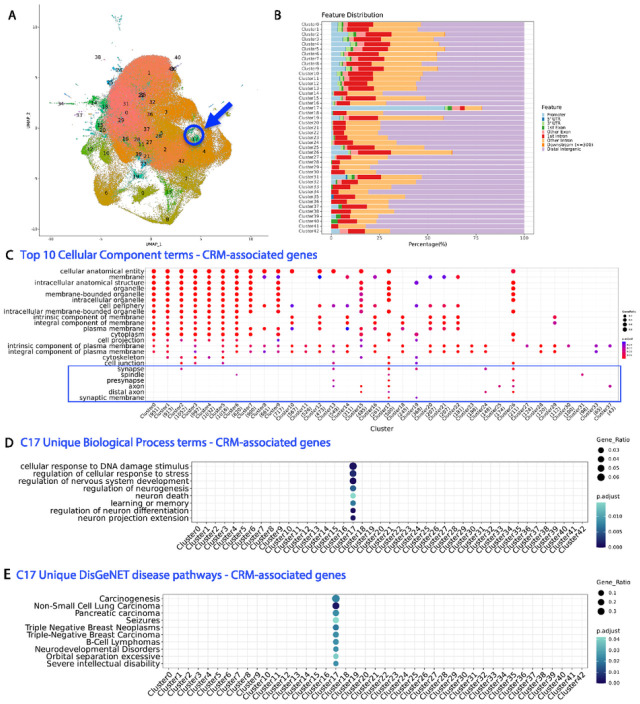
Unsupervised machine learning for cluster annotation. **A)** Mouse chr17 UMAP identified a total of 43 CRM clusters. Each CRM cluster represents a set of CRMs with similar transcription factor binding site (TFBS) compositions. The highlighted region (blue arrow) represents cluster 17 (C17). **B**) Genomic annotation and feature distribution of the 43 clusters. **C**) Top 10 gene ontology (GO) cellular component (CC) significant terms on the CRM-associated genes of the 43 clusters. Clusters not shown in the dot plot did not have any significant pathway enrichment in this category. **D)** Significant Biological Process (BP) terms uniquely enriched in C17-associated genes. **E**) Significant DisGeNET disease-associated genes uniquely enriched in C17-associated genes.

Next, we sought to understand the regulatory code encoded in the CRMs by determining the tissue, cell type, developmental stage, or stimulus response-specificity of each CRM. We first scanned the mouse genome for TFBS using PWMs of known TFs from the CIS-BP database. We obtained TFBS abundance in each CRM for every TF in the CIS-BP database and performed unsupervised clustering using single-cell genomics tool Seurat^51^. Due to the large dataset and limitations of computational infrastructure to complete analysis on creating the Seurat object for each chromosome, we narrowed our analysis of the smaller mouse chromosome 17 as a test case. We obtained a total of 43 CRM clusters (Fig.5A). Each CRM cluster represents a set of CRMs with similar TFBS compositions. Cluster marker genes represent TFs whose binding sites are enriched in the CRM cluster. Genes associated with the CRMs in the cluster by proximity (distance to TSS) represent the putative target genes. Therefore, each CRM cluster linked putative TFs with their target genes through component TFBSs located within the CRMs. Most of the clusters included CRMs that were located in the distal intergenic and intronic regions, with the exception of cluster #17 (referred to as C17 herein), which enriched for CRMs located in the promoter regions (Fig. 5B).

To investigate the functionalities of the clusters of CRMs, we performed pathway enrichment analyses on the CRM-associated genes of the 43 clusters. Most clusters were enriched for some cellular component pathways with C17-associated genes enriched for multiple pathways involved synapse and axon components (Fig.5C, Supplementary Table 7). Many clusters uniquely enriched for different Biological Process pathways (Supplementary Fig. 2A) suggesting that they could regulate genes involved in distinct biological processes. Multiple pathways including regulation of nervous system development, regulation of neurogenesis, learning or memory, neuron projection extension, neuron death, were uniquely enriched in C17-associated genes (Fig. 5D) suggesting their nervous system related functions. Cellular response to DNA damage stimulus and regulation of cellular response to stress were also uniquely enriched in C17-associated genes (Fig. 5D). Six clusters had signi cant enrichment of KEGG pathways including C17 with multiple pathways in cancer and focal adhesion, which is important for axonal branching and synapse formation^52^ (Supplementary Fig.2B, Supplementary Table 8). Additionally, using DisGeNET, a database of genes and variants associated to human diseases^53^ we found that CRM-associated genes from multiple clusters, including C17, were enriched for cancer and autism spectrum disorder (ASD) (Supplementary Fig.2C, Supplementary Table 9). Notably, C17-associated genes were uniquely enriched for multiple cancer pathways, neurodevelopmental disorders, and severe intellectual disability using DisGeNET database in ClusterProfiler (Fig. 5E).

**Figure 6:**
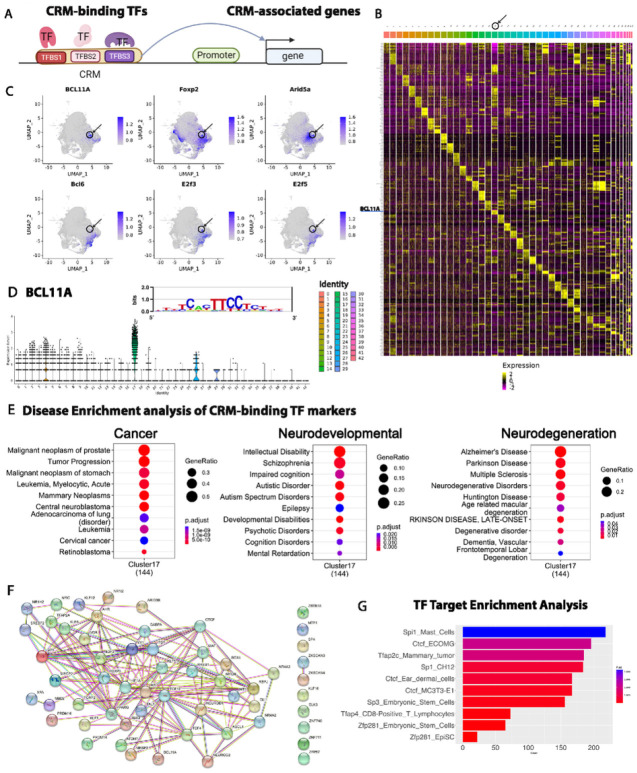
Multiple lines of evidence supporting the link between the CRMs with their binding TFs and target genes. **A**) Schematic showing that CRMs link the putative regulatory TFs (CRM-binding TFs) with their putative regulated targets (CRMs-associated genes). **B**) Heatmap with top 10 CRM-binding TF marker genes for each cluster. **C**) Feature plot of CRM-binding TF markers of C17 with a black circle and arrow representing C17 in the UMAP. **D**) Violin plot of BCL11A marker showing its higher abundance in C17 and the BCL11A binding motif (M07926_2.00) from CIS-BP database. **E**) Disease enrichment analysis of TF markers enriched for cancer, neurodevelopmental disorders, and neurodegeneration. **F**)STRING network showing that TF markers interact with each other. **G**) Top 10 TFs and tissues/cell types from ChIP-Atlas that enriched for C17 CRM-associated genes.

Next, we investigated the functions of the CRM clusters by examining the cluster marker TFs (Fig. 6A, CRM-binding TFs). Each cluster had a set of TFs that were highly enriched in the cluster although most of the time not uniquely (Fig. 6B–D). Due to the unique association of C17 with cancer and neurodevelopmental disorders we investigated it in further detail and uncovered multiple lines of evidence (Fig. 6C–G) connecting the CRMs with their binding TFs (C17 marker genes, Supplementary Table 10) and target genes (C17-associated genes, Supplementary Table 11) consistent with their function in tumorigenesis and neurodevelopment. First, many of the binding TFs are known cancer drivers such as Ascl1^54^, Bcl6^55^, Atoh1^56^, Arid5^57^, and multiple E2F family TFs^58^ (Fig. 6B). They were significantly enriched for various cancer pathways (Fig. 6E, Supplementary Table 12) as determined by three different pathway enrichment tools using DisGeNET database: disgenet2r, ClusterProfiler, and EnrichR. Similarly, many of the binding TFs are known regulators of neurodevelopment and neurodegeneration diseases and are significantly enriched for neurodevelopmental disorders and neurodegenerative pathways (Fig. 6E, Supplementary Table 12). For example, Foxp2^59^, Bcl11a^60^, Hey1^61^, and Tcf4^62^ (Fig. 6B) have been linked to ASD, schizophrenia, and intellectual disability whereas astrocytic Cebpd^63^ contributes to the progression of Alzheimer’s Disease. Additionally, top C17 CRM-binding TFs known to cooperatively regulate target gene expression (Fig. 6F) such as TCF4 that interacts with ASCL1 and NEUROD1^64^. Furthermore, many C17 CRM-binding TFs that play important roles in neurodevelopment have been linked to glioblastoma and brain tumors such as ASCL1^54^, BCL6^55^, Atoh1^56^, and Arid5^57^, consistent with the known link between neurodevelopmental pathways and brain tumor^56^. CRM-binding TFs were also enriched in embryonic expression and pluripotent stem cells by STRING functional enrichment analysis (Supplementary Fig.2D), consistent with the knowledge that most ASD risk genes regulate gene expression or to be involved in neuronal communication during early brain development^65^. Many of the enriched pathways were shared by the CRM-binding TFs and the C17 CRM-associated genes (Supplementary Fig.2D-E) linking the putative TF regulators with their targets.

To test whether C17- associated genes are the biological targets of the C17 CRM-binding TFs we tested the overlap between these genes with the known TF target genes in the ChIP-Atlas database^32^ defined by the mouse ChIP-seq experiments. C17-associated genes were enriched in 149 different combinations of TFs, cell lines, and tissues for 21 of the C17 CRM-binding TFs such as Ascl1, CTCF, Tfap2c, and Neurod1 (Fig. 6G, Supplementary Table 13). Multiple sets of CTCF targets were enriched for the C17-associated genes including targets determined in the brain and cerebellum. CTCF dosage deficiencies has been linked to developmental delay and intellectual disability^66^. The enrichment of Tfap2c targets in mammary tumor and Maz targets in murine erythroleukemia cell suggesting that the CRM-associated genes represent biological-relevant targets of the CRM-binding TFs. Taken together, these results provide strong evidence that CRMs in C17 linked CRM-binding TFs with their targets in regulating cancer, neurodevelopmental and neurodegenerative diseases.

### Using CRM to guide experimental design

**Figure 7:**
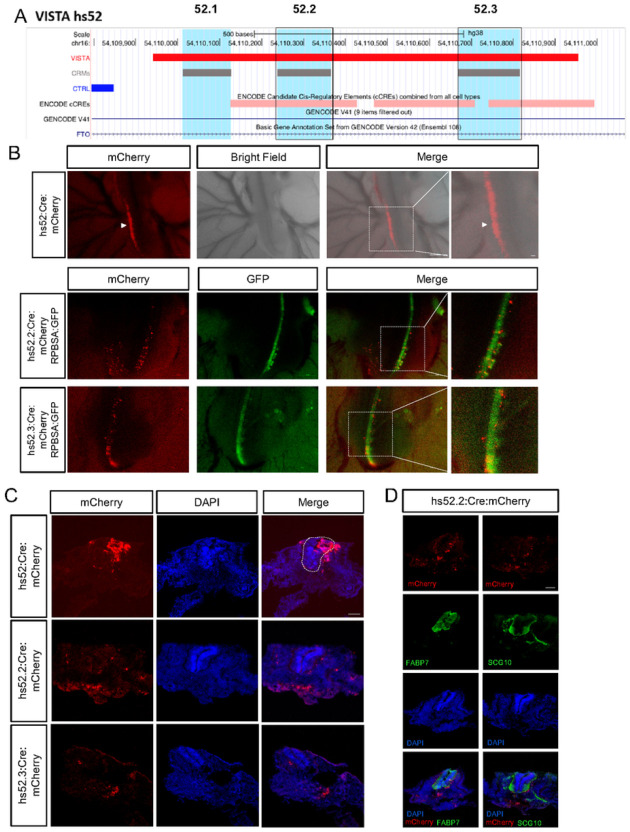
*In-ovo* characterization of VISTA enhancer and predicted CRMs through electroporation and live imaging. **A**) Schematic of the VISTA enhancer hs52 and the predicted CRMs on human chromosome 16. **B**) VISTA hs52 and the CRMs hs52.2 and hs52.3 enhancers driving mCherry expression in a whole mount view in chick embryo 24 hour after electroporation. RPBSA:GFP plasmid was co-electroporated. Arrow heads- neural tube. n= 5 independent animals per enhancer. Scale 1000μm, zoomed image- 100μm. **C**) Transverse sections of electroporated embryos with enhancers driving mCherry and co-stained with DAPI. Dashed line-neural tube. n= 5 independent animals per enhancer. Scale 100μm **D**) Immunofluorescence of the CRM hs52.2 (red) and the glia marker FABP7 or the neuronal marker SCG10 (green) along with DAPI (blue). n= 4 independent animals. Scale 100μm.

Many VISTA enhancers drive reporter gene expression in multiple tissues or brain regions therefore likely contain multiple enhancers as well as non-functional sequences between those enhancers. We used our predicted CRMs to guide experimental design to delineate enhancers that drive more restricted expression patterns through more detailed analysis. To validate the predicted CRM function compared to the original VISTA enhancer, we tested their ability in driving reporter gene expression in chick embryo neural crest cells. During embryonic development, every cell differentiates and becomes specialized to assemble an organ or a physiological system. Therefore, tracing the fates of specific cells provides important understandings for monitoring organogenesis, physiological, and pathological processes. Despite recent success in cataloging the gene expression profiles of distinct cell subpopulations, there is still limited ability to specifically access subpopulations to study their function. Neural crest cells serve as a great model for cell differentiation as they are progenitor cells, located at the embryonic dorsal neural tube, that differentiate into many derivatives^67^. These derivatives range from peripheral nervous system components such as sensory and autonomic neurons, satellite glia and Schwann cells, to endocrine cells and melanocytes^68^. Mouse and human enhancer elements have been shown to be active in the chick neural tube^69,70^. The accessibility of the embryonic chick neural tube, together with the evolutionary conservation of many human and mouse enhancer elements suggested the chick embryo as an ideal model system for deciphering neural crest cells differentiation. VISTA Enhancer #52 (hs52, 1013bp) flanking the genes Fto-It1 and Irx3 on human chromosome 16 was identified through a transgenic mouse screens of highly conserved non-coding sequences in the human genome to drive β-galactosidase reporter gene expression at embryonic day (E) 11.5 in the dorsal root ganglion (DRG) and trigeminal ganglions. Three predicted CRMs overlapped the hs52 enhancer: 52.1 (116bp), 52.2 (127bp) and 52.3 (147bp) (Fig. 7A). We chose to test elements hs52.2 and 52.3 because they overlapped also with ENCODE cCREs (Fig. 7A). Elements hs52, hs52.2 and hs52.3 were PCR amplified from human genomic DNA and cloned upstream to a Cre recombinase. To verify the specificity of the expression, the Enhancers::Cre plasmids were electroporated into stage 16 to 17 chick hemi-tube along with a Cre-dependent mCherry plasmid [pCAGG-LoxP-STOP-LoxP-mCherry]. Green fluorescent protein (GFP) driven by the general synthetic promoter RPBSA, was used as a control for efficiency of the electroporation (Fig.7B). While hs52 element showed wide expression in many cells along the embryo neural tube, the elements hs52.2 and hs52.3 showed a more specialized expression (Fig. 7B). Transverse sections along the embryo further demonstrated that hs52 drives expression in cells inside the neural tube, as well as cells migrating outside of the tube, whereas elements hs52.2 and hs52.3 drive mCherry expression in more specialized population of cells migrating outside of the neural tube (Fig. 7C). These results suggest that our analysis defined sub-elements of hs52 enhancer that contribute to common expression control in an additive way. To further decipher the identity of the specified cells, driven by hs52.2 element, co-staining with the glia marker FABP7 and the neuronal markers SCG10/STMN2 was performed (Fig.7D). Most cells did not show co-localization with the glia or neuronal marker, therefore their fate might be endocrine or pigment cells, or they still have not completed their differentiation. Nevertheless, this experimental validation is sufficient to confirm the hypothesis that our predicted CRMs are potentially functional and can drive more restricted gene expression.

## Discussion

CRMs are still poorly annotated^71^ and their discovery has been challenging^1^. We developed MrMOD, an algorithm to predict CRMs in mammalian genomes. Extensive comparison of predicted functional CRMs and predicted non-functional regions in the genome with curated ExpCRMs and large collections of epigenomic datasets demonstrated high sensitivity and accuracy. Dissection of a VISTA enhancer to define smaller enhancers that drive more cell-restricted expression with the guide of our predicted CRMs demonstrates the value of the predicted CRMs. Thus our CRM predictions will have wide applications and broad impact on transcription regulation research in evolution and development.

How many regulatory elements are there in mammalian genomes? Currently there is a wide range of estimates of the number of regulatory elements in mammalian genomes ranging from ~ 1.47 million CRMs covering about 55% of the human genome^72^ to more than 3 million, covering 10–80% of the non-coding human genome^73^. The ReMap 2022 database reported 2.4 million and 3.4 million non-redundant CRMs in their mouse and human regulatory atlases, respectively^74^. The Enhancer Atlas 2.0 database obtained more than 700,000 predicted enhancers across mouse and human species^21^. The accessible chromatin landscape of human genome identified approximately 2.9 million DHSs through genome-wide profiling in 125 tissues/cell types^14^. Mouse scATAC-seq brain atlas identified 491,818 elements from 45 brain regions and 160 cell types in adult mouse cerebrum alone^24^. Human scATAC-seq data provided ~ 1.2 million elements from 45 adult/fetal issues and 222 cell types^25^. Given the limitation of how many datasets are currently available, our prediction of ~ 5.5 million (44.1% genome coverage) and 6.1 million (42% genome coverage) CRMs for the mouse and human genomes, respectively, is a reasonable upper estimate of the regulatory universe of these genomes.

It has been pointed out that the majority of putative elements identified by empirical methods are merely predictive, and biological validation is essential before assigning a definitive regulatory function to a genomic region^1^. Experimentally defined CRMs remain the gold standards of functional CRMs. However, due to the low-throughput, expensive, and time-consuming nature of this validation approach there are very limited data available. VISTA Enhancer Browser, the only database that has a collection of experimentally validated mammalian enhancers, covers only ~ 0.05% of the mouse and human genomes. Our literature-curated 157 experimentally defined CRMs that have minimal sequence sufficient for function and are not available on VISTA, expanded the current database by 9.6%. Our computational and experimental validation of predicted CRMs demonstrate high sensitivity and accuracy of our prediction. Despite the large number of available experimental datasets collected, we emphasize that we still don’t have all the possible experimental data for every tissue, every cell type, and every developmental stage, or stimulation condition. Therefore, our work here fills in a critical gap in knowledge by providing a comprehensive base-pair resolution annotation of the functional and non-functional elements in mammalian genomes.

Our study has a potential impact on the research of transcription regulation and could revolutionize the way in which epigenomic data are analyzed. A crucial first step in analyzing epigenomic data such as ChIP-seq, ATAC-seq, and DHS-seq data pertains to finding peaks that correspond to targeted DNA regions. The numbers and the boundaries of peaks called for in the same sample depends heavily on the specific parameter settings and the algorithm used as well as the sequencing depth and the quality of the experiment itself^75^. Different peak boundaries or presence/absence of peaks in different replicates are common problems^19,75^. Many tools have been developed trying to define a consensus region from peaks overlapping among a set of replicates with varied exact positions, but different tools give very different consensus peaks, with differing lengths and positions^19^. These differences are sufficient to influence downstream biological interpretations, and lead to disparate scientific conclusions about enhancer biology and disease mechanisms^76^. Our predicted CRMs could instead serve as a reference (refCRM) to map reads directly to those reference CRMs similar to mapping reads to gene models in RNA-seq analysis. Therefore, data from different replicates and different conditions would have signal information within the exact same genomic locations, enabling direct comparison across samples and conditions as well as the identification of differential CRM activity, similar to differentially expressed gene identification. Second, our work provides a valuable resource for guiding experimental design. Because our computational prediction is independent of the epigenomic data used for evaluating our predicted CRMs, the combination of these two orthogonal types of information will provide strong support for the functionality of a genomic region to guide experiments to dissect gene regulation as demonstrated in our current study. We provide here to the research community the genomic locations of all predicted CRMs ranked by the number of lines of supporting evidence. Furthermore, the small size (average ~ 200 bp) of predicted CRMs is ideal for MPRA assays because most studies synthesize libraries of candidate enhancers on microarrays, generally at ~ 200 bp^77,78^. Third, genome-wide association studies (GWAS) have identified many non-coding variants that are in linkage disequilibrium with the causal variant but have not been able to pinpoint the causal variants in general. Our refCRMs could help to search for likely causal variants and to elucidate molecular mechanisms that support the genetic bases of diseases and complex traits in mammalian species. Fourth, the use of unsupervised machine learning allows the identification and annotation of clusters of CRMs with similar functionality, define TF markers and CRM targets, CRM-motif composition, and potential cooperation between TFs in the clusters. This information contributes to the understanding of gene regulatory networks. Based on the evidence collected, we successfully identified a cluster that is linked to neurodevelopmental disorders, cancer, and neurodegeneration. Lastly, our work will enable a systematic evaluation of CRM evolution and CRM activity regulation during development, evolution, environmental stimulation, and disease pathogenesis. Our PWM identification was based on the genomic sequences of mouse, rat, and human, and the detected PWMs are evolutionarily conserved among the species. Because MrMOD uses the set of mammalian-genome-specific PWMs to predict candidate CRMs, we expect MrMOD to be applicable to any species that evolutionarily resides between mouse and human within the mammalian phylogenetic tree, including non-human primate where experimental compendiums are not generated and it would be costly to do so. However, MrMOD does not require conservation information at CRM prediction step and should be able to detect non-conserved CRMs.

## Methods

### Genome sequence and orthologous genes

The unmasked genomic DNA sequences and annotation of mouse (*Mus musculus*) assembly GRCm38 (mm10) and human (*Homo sapiens*) assembly GRCh38 (hg38) from the Genome Reference Consortium were downloaded from the Ensembl website (https://nov2020.archive.ensembl.org/Mus_musculus/Info/Index and https://useast.ensembl.org/Homo_sapiens/Info/Index). Mouse genome was used as the anchor genome. Human and rat orthologs of mouse genes were downloaded from BioMart (https://useast.ensembl.org/info/data/biomart/index.html). Intergenic region sequences of up to 5 kb in length upstream of the start codon ATG of each gene in the corresponding genome were retrieved. If the distance to the next upstream gene is less than 5 kb, only the intergenic region was obtained.

### PWM prediction using PhyloNet and motif consolidation

Using mouse genome as the anchor, we obtained a set of 18,215 genes with at least one ortholog in the rat or human genome. We retrieved up to 5 kb sequences upstream of the ATG codon for all of the genes. Each mouse gene and its orthologs formed a data entry and was used as input by PhyloNet^28^ to query the database, which includes all the orthologous gene sets to identify conserved cis-regulatory elements that were associated with multiple genes. PhyloNet was run with options “-q 2000 -iq 500 -id 500 -s 4 -c1 -o2 -pf 50”. Up to 50 of the most significant PWMs from each query gene set were saved for further analysis. Because these initial predicted motifs in the PhyloNet output files are highly redundant, we took two steps to consolidate predicted motifs using the ALLR statistics^31^ as described previously^29^. Briefly, the first step compares matrices in each query output file to consolidate matrices obtained from the same query sequences that significantly overlap. The unique PWMs obtained from each query gene at the first step were pooled together and further consolidated to generate the final set of 5,143 distinct motifs (p-value < 10^−10^) with lengths between 5 and 30 bases (average 18 bp).

### Comparison with transcription factor PWMs in the TRANSFAC and CIS-BP database

Predicted PWMs were compared with PWMs of CIS-BP (Version 2.00) and TRANSFAC (version 10.2)^27,79^ using MatAlign-v4a (Wang and Stormo; http://stormo.wustl.edu/MatAlign/). Matrices that had an ALLR score > 6.57 and the percentage of overlap between two matrices (OLAP score) > 68.1% were considered redundant^29^. For each round of comparison, the best PWM was picked rst (the one with the highest total ALLR score in the PhyloNet output). It was compared with the rest of the matrices using ALLR statistics, and any matrix that appeared redundant to the chosen matrix was removed. Then, the second best one was picked, and the process was repeated until all the matrices had been analyzed.

### ChIP-Atlas database mouse transcription factor target enrichment analysis

ChIP-Atlas^32^ has a collection of analysis results from 2,540 ChIP-seq data sets including TFs and their potential target genes, totaling 723 TFs from 369 different cell types/tissues for mouse (mm10) (ftp://ftp.biosciencedbc.jp/archive/chip-atlas/data/mm10/target). The peaks used in this study from ChIP-Atlas were +/− 5kb from the transcription start site (TSS) of RefSeq. The threshold of significance used to determine whether a target gene is enriched for a TF was > 500 binding scores of MACS2. Significant overlap between predicted PWM-associated genes and TF targets from ChIP-Atlas were evaluated using a hypergeometric test. FDR-corrected p-value < 0.05 was considered significant.

### Functional enrichment analysis of PWM-associated genes

Each PWM-associated gene set was compared with GO and KEGG annotation using the *ClusterProfiler* R package^80^. Terms and pathways with FDR corrected q-value < 0.001 were considered significant.

### Whole-genome-wide CRM identification

The 5,143 mammalian PWMs identified by PhyloNet were used as input for the algorithm implemented in CerMOD^29^ to identify CRMs in the mouse and human genomes, respectively. First, Patser^81^ was used to identify all predicted binding sites for the 5,143 PWMs using default cutoff scores. Next, the algorithm calculates the average number of binding sites per position in each chromosome and Z score for each position. Peak positions that have a Z score > 2.33 (corresponding to p-value < 0.01, one-tailed) were identi ed. For each peak position, we extended it in both 5’ and 3’ directions if the next Z score > 0 position is fewer than 30 bp away (the longest motif length). Peak positions used in a previous extension step were not extended.

MrMOD identifies DNA regions that have TF binding sites significantly more than average (motif abundance z score > 2.33, p < 0.01, one-tailed) and automatically determines the boundaries by z score = 0 positions at each end of the DNA region. These regions correspond to putative regulatory CRMs.

### Control regions development and annotation

To develop a set of control regions from each genome with the exact same genomic coverage and distribution, we first obtained regions not covered by CRMfull using BEDTools^82^ *complement* function excluding regions with more than 50% “Ns”. Because it is impossible to predict the exact boundaries of CRMs, regions smaller than 400 bp that were located between two predicted CRMs, were filtered out to remove small regions that could be part of the nearby CRMs. Next, we defined a subset of CRMs (CRMsub) that are smaller than 250bp as input. Then we trimmed the control regions by 40 bp each end, padding 5bp between fragments, to generate a distribution with exactly the same number/length as the CRMsub. The R package ChIPseeker^83^ was used to annotate CRMfull and control regions as well as calculate feature genomic distribution.

First, regions not covered by CRMfull were obtained. Then, we subset the CRMs by eliminating the longest CRMs, resulting in predicted CRM subsets (CRMsub) that are smaller than 250 bp. Because of the lack of space to sample the control region, using small fragments gives sufficient space to sample the non-functional regions to get the control with the exact same number/length as the subset. In addition, it is impossible to predict the exact boundaries of CRMs. For this reason, small regions that were located between two predicted CRMs were filtered out as they could be part of the nearby functional CRMs. These regions were predicted to be non-functional, covering 21.8% and 20.8% of mouse and human genomes, respectively, and were used as the control regions (referred to as CTRLs) to evaluate the accuracy of predicted CRMs. CRMsub had the exact same genomic coverage and distributions relative to annotated genes and transcripts to the CTRLs for both genomes (Fig. 2B, 2C).

### Comparison with experimentally defined CRMs, chromatin accessibility, and epigenomics data

Experimentally defined modules came from two sources. 1) We collected 97 experimentally defined CRMs for mouse and 60 modules for human through an extensive literature search (Supplementary Tables 1 and 2). 2) We downloaded 634 and 998 experimentally defined enhancers that exhibited enhancer activity^10^ from VISTA Enhancer Browser for mouse and human, respectively.Human and mouse predicted enhancers were obtained from Enhancer Atlas 2.0 (http://www.enhanceratlas.org/data/download/species_enh_csv.tar.gz). From the Cistrome database, chromatin accessibility (ATAC-seq and DNAse-seq) peaks and ChIP-seq histone marks (H3K27ac, H3K4me1, and H3K4me3) peaks were downloaded (http://cistrome.org/db/batchdata) for both mouse and human genomes. ATAC-seq Atlas is only available for mouse genome^50^. ENCODE mouse and human cCREs^22^ are available at the web-based server Search Candidate cis-Regulatory Elements by ENCODE V3 (SCREEN; http://screen.encodeproject.org). Mouse^24^ and human^25^ scATAC-seq can be accessed at their portal (http://catlas.org/mousebrain/#!/ and http://catlas.org/humanenhancer/#!/, respectively). Each dataset included peaks from hundreds of different tissues and cell types. Files from the same type of data were merged using *bedtools* merge to eliminate overlapping elements. Modules bigger than 2.5 kb after merge were eliminated before comparison. We define an experimentally defined module as being correctly predicted at two different cutoffs: 1) experimentally defined modules overlap with predicted CRMs by 50% of the length of the shorter one; 2) they overlap by 1 bp. The odds ratio (OR), confidence interval, and p-value (chi-square independent test) were calculated comparing CRMsub and CTRL of each dataset of mouse and human. Peak-detection sensitivity and OR were calculated using the R package *fmsb*. The difference was considerate significant with an OR > 1 and p-value < 0.05.

### Unsupervised machine learning to annotate CRM functions

We scanned the mouse genome for TFBS using mouse and human PWMs from the CIS-BP database. We obtained TFBS abundance in each CRM for every transcription factor in the CIS-BP database. Unsupervised clustering of CRMs based on TFBS abundance using single-cell genomics tool Seurat on chromosome 17, one of the smallest chromosome of the mouse genome. Each CRM cluster linked putative TFs (TF marker genes whose binding sites are enriched in the CRM cluster) with the putative target genes (genes associated with the CRMs in the cluster). The CRM associated-genes of each cluster were annotated using ChIPseeker R package.

### Functional enrichment analysis of all CRM associated-genes

The annotated chr17 CRM associated-genes of each cluster were used to perform functional enrichment analysis: GO, KEGG, and DisGenet enrichments using ClusterProfiler R package. For DisGenet enrichment analysis, the mouse ENTREZ IDs were converted to human ENTREZ IDs. DisGenet was also tested with two other methods (EnrichR and disgenet2r) to compare and ensure the results of C17. Parameters used in the ClusterProfiler functional enrichment analysis were FDR < 0.05, minGSSize = 10, maxGSSize = 5000, minimum number of counts in a pathway = 5. Genes of chromosome 17 (chr17) were used as universe background for these analyses.

### Functional enrichment and network analysis of TF markers of C17

The TF markers of C17 were also enriched using the same parameters described above. The only difference is that we did not include chr17 as universe, because TFs are not restricted to bind only CRMs of chr17. TFs that were enriched in three neurodevelopmental pathways (intellectual disability, neurodevelopmental disorder, autistic disorder, and schizophrenia) were combined (total of 57 TFs) and used to build a STRING network to visualize the connections between them.

### CRM associated-genes of C17 are enriched for mouse ChIP-Atlas TF targets

TF targets enrichment analysis was performed using the same previous parameters used for the motif analysis: +/− 5kb from the TSS of RefSeq; threshold of significance used to determine whether a target genes is enriched for a TF was > 500 binding scores of MACS2; significant overlap between predicted CRM associated-genes of C17 and mouse TF targets from ChIP-Atlas were evaluated using hypergeometric test. FDR-corrected p-value < 0.05 was considered as significant.

### Animals and procedures

Fertilized White Leghorn chicken eggs (Hy-line North America, Mansfield, GA) were incubated at 38.5–39 C and 50–80% humidity. A DNA solution of 2–5 mg/ml was injected into the lumen of the neural tube at HH stage 17–18 (E2.75-E3). Electroporation was performed using 3 × 50 ms pulses at 25 V, applied across the embryo using a 0.5-mm tungsten wire and a BTX electroporator (ECM 830). 100 unit/ml penicillin in Hank’s Balanced Salt Solution was added on top of the embryos and embryos were incubated for 24 hours prior to analysis.

### Immunohistochemistry

Embryos were fixed overnight at 4 C in 4% paraformaldehyde/0.1 M phosphate buffer, incubated in 30% sucrose/PBS for 24 h, and embedded in Optimal Cutting Temperature (O.C.T.). Cryostat sections (12 μm) were collected on Superfrost Plus slides and kept at −20 C. The following primary antibodies were used- rabbit anti FABP7 (Thermo Fisher Scientific Cat #PA5–24949) and rabbit anti SCG10/STMN2 (Novus catalog #NBP1–49461). Secondary antibody used-647 goat (Thermo Fisher #A-21245). Images were taken under a microscope (SMZ-745T Zoom Stereo Photo Microscope, Nikon and Lionheart LX automated microscope, BioTek) and images were analyzed with Nikon NIS-Elements and Gen5 software.

### DNA

The hs52 element was amplified by PCR from a genomic human DNA utilizing the primers [GCCAATTGCAATTTGGAATAACTTTCCCTACCC] and [GCGCTAGCTAAAAAGTGACCTGGGAAAACTCAG]. hs52.2 element was amplified utilizing the primers [GCCAATTGAACGCACCCTCTGTTCTTCAGT] and [GCGCTAGCGCACTAAGTACTATTATGTAGCACA]. hs52.3 element was amplified utilizing the primers [GCCAATTGCAGGCTTGGAAATGGGGCCAGG] and [GCGCTAGCACGTGGCAGTGAAAACGAGTGG]. The enhancers were cloned into 5’MfeI/3’NheI sites of the Cre plasmid. RPBSA: GFP plasmid used as positive control for electroporation (Addgene #60511).

hs52

CAATTTGGAATAACTTTCCCTACCCagtaaattgagcattactctaggattctgagacagagagaaagcacaattttaaaagctttgcagagttcctttgtaattagtcgcagctttccttgaatattaattttccctgca

hs52.1

TACCCAGTAAATTGAGCATTACTCTAGGATTCTGAGACAGAGAGAAAGCACAATTTTAAAAGCTTTGCAGAGTTCCTTTGTAATTAGTCGCAGCTTTCCTTGAATATTAAT

hs52.2

CAGAACGCACCCTCTGTTCTTCAGTGCAGTGTGTAGCTTATCAGTGCAAACAGTTTAATATTTATGCTAAGAGGATTGTCAAAAGCAGCTTCTGTTGCTTTAATTCTTGTT

hs52.3

CCTCTCTACCATCAGGCTTGGAAATGGGGCCAGGATATTCCATTCTTTGATCTCTTCATAGTCAGTCCTACACAGTCAGAAGACAAATAGTGAGCATGACCACTTTTTAAT

## Data Availability

The code and all relevant data have been submitted to the WashU Epigenome Browser and can be visualized at the following URLs: Human: https://epigenomegateway.wustl.edu/browser/?sessionFile=https://wangftp.wustl.edu/~dli/gzhao/CRMs-202207/hg38-s.json Mouse: https://epigenomegateway.wustl.edu/browser/?sessionFile=https://wangftp.wustl.edu/~dli/gzhao/CRMs-202207/mm10-s.json
